# Supplementary material for: A Qualitative Meta-Synthesis of Studies on Workplace Bullying among Nurses
Source: Int J Environ Res Public Health. 2022 Oct 29;19(21):14120. doi: 10.3390/ijerph192114120 (PMC9659011; doi:10.3390/ijerph192114120)
Supplement: Supplementary file 1 [file ijerph-19-14120-s001.zip › Table S1_R_Final.pdf]

Table S1. List of studies analyzed and synthesized in this study

ID	Authors	year	Title	Journal	Purpose	Analytic methods	Participants	Data collection methods
INT1	J. E. F. Arnetz, L. Cotten, S. R. Jodoin, C. Chang, C. D. /U.S.A	2019	Workplace Bullying Among Nurses: Developing a Model for Intervention	Violence Vict 34(2): 346-362	to explore experiences of bullying and ideas for interventions to prevent bullying among a sample of RNs	content analysis	4 focus groups, 15 nurses (3 groups of 11 staff nurses, 1 group of 3 NMs, all female)	focus group discussion
INT2	P. S. H. Autrey, J. L. Wech, B. A. /U.S.A	2013	Sources, reactions, and tactics used by RNs to address aggression in an acute care hospital: A qualitative analysis	Journal of Nursing Administration 43(3): 155-159	to (a) identify the sources of aggression that nurses face in the workplace, (b) determine how nurses react to aggression in the workplace, and (c) identify tactics that nurses use to address aggression in the workplace	grounded theory	47 RNs (91% female, 9% male) with an average of 17 years of clinical experience	semi-structured interviews<br><br>(interviews with 15 NMs were conducted prior to those with staff nurses)
INT3	G. O. A. Boateng, Tracey L. /Canada	2016	'Drop dead… I need your job': An exploratory study of intra-professional conflict amongst nurses in two Ontario cities	Social Science & Medicine 155: 35-42	to explore the experiences of visible minority and White nurses, including the conflicts they experience in practice	qualitative research design	66 RNs and RPNs (6 males, 60 females/28 White, 38 visible minorities)	semi-structured one-on-one in-depth interviews
INT4	A. M. D. Castellón /Cille	2011	Occupational Violence in Nursing: Explanations and Coping Strategies	Revista Latino-Americana de Enfermagem (RLAE) 19(1): 156-163	to explore the explanations of harassment given by nurses who were victims of it and how they are related to their coping strategies to formulate a comprehensive model that accounts for the elements involved and that can be used to develop prevention strategies	grounded theory	13 Chilean nurses with an average of 3.9 years of clinical experience (average length of harassment was 19 months (2–84 months))	semi-structured interviews
INT5	N. F. D'Souza, D. Tappin, D. Catley, B. /New Zealand	2018	Conceptualizing workplace cyberbullying: Toward a definition for research and practice in nursing	Journal of nursing management 26(7): 842-850	to conceptualize workplace cyberbullying through two research objectives:<br>(1) to explore how the group of interest, nurses in New Zealand, understands the issue of workplace cyberbullying and<br>(2) to develop a definition of “workplace cyberbullying” in nursing that could be used in the	thematic analysis	16 nurses (14 female, 2 male)<br>2/3 under 35 years old	semi-structured interviews

					management of cyberbullying as well as in future research			
INT6	H. H. Ebrahimi, Hadi Negarandeh, Reza Jeffrey, Carol Azizi, Azim /Iran	2017	Violence against new graduated nurses in clinical settings: A qualitative study	Nursing Ethics 24(6): 704-715	to understand Iranian experienced nurses' use of lateral and horizontal violence against new graduate nurses (NGNs)	conventional content analysis	18 experienced nurses	unstructured and semi-structured interviews
INT7	J. B. Evans, E. Sanber, S. /Australia	2008	The strengths and weaknesses of transitional support programs for newly registered nurses	Australian Journal of Advanced Nursing 25(4): 16-22	to determine the strengths and weaknesses of transition support programs for newly registered nurses	theme extraction ("pile on the kitchen table" method by Roberts and Taylor)	9 newly graduated RNs and 13 experienced RNs	semi-structured interviews
INT8	D. A. D. Gaffney, R. F. Hofmeyer, A. Vessey, J. A. Budin, W. C. /U.S.A	2012	Making things right: nurses' experiences with workplace bullying-a grounded theory	Nurs Res Pract 2012: 243210	to understand how nurses encounter bullying in the workplace and the strategies they use to protect themselves and their patients	constructivist grounded theory	81 nurses	narratives for open-ended question embedded in an online survey
INT9	M. Griffin /U.S.A	2004	Teaching Cognitive Rehearsal as a Shield for Lateral Violence: An Intervention for Newly Licensed Nurses	Journal of Continuing Education in Nursing 35(6): 257-263	to (1) provide a theoretical basis for understanding the origins and manifestations of lateral violence in nursing, (2) identify and acknowledge the vulnerability of newly registered nurses, and (3) provide instruction on the use of cognitive rehearsal	exploratory design with applied intervention	26 newly licensed nurses who were taught about lateral violence and the use of cognitive rehearsal techniques	videotaped focus groups designed to collect qualitative data about the applied intervention
INT10	M. V. Hutchinson, Margaret H. Jackson, Debra Wilkes, Lesley /Australia	2005	I'm gonna do what i wanna do. Organizational change as a legitimized vehicle for bullies	Health Care Management Review 30(4): 331-336	to explore the use of processes of organizational change as a vehicle for bullying	constant comparative method	26 nurses who had personal experience of workplace bullying (WPB**)**	in-depth, semi-structured qualitative interviews
INT11	M. V. Hutchinson, M. H. Jackson, D. Wilkes, L. /Australia	2006	'They stand you in a corner; you are not to speak': nurses tell of abusive indoctrination in work teams dominated by	Contemporary Nurse: A Journal for the Australian Nursing Profession 21(2): 228-238	to depict bullies who worked together in alliances	constant comparative method	26 nurses	in-depth, semi-structured interviews

			bullies					
INT12	M. V. Hutchinson, Margaret H. Jackson, Debra Wilkes, Lesley /Australia	2006	Like wolves in a pack: Predatory alliances of bullies in nursing	Journal of Management & Organization 12(3): 235-250	exploring nurses' experiences of being bullied as well as their beliefs, understandings, and perceptions of bullying and why it took place<br>research questions:<br>(a) What was the nature of the bullying?<br>(b) What were the effects of these bullying experiences?<br>(c) What were the nurses' perceptions of bullying?<br>(d) What strategies or action did the nurses take? and<br>(e) What were the nurses' perceptions of organizational policies and procedures on bullying?	constant comparative analysis	26 nurses who had experienced WPB from 2 hospitals	individual in-depth, semi-structured interviews
INT13	M. V. Hutchinson, Margaret H. Wilkes, Lesley Jackson, Debra /Australia	2009	'The worse you behave, the more you seem to be rewarded': Bullying in nursing as organizational corruption	Employee Responsibilities and Rights Journal 21(3): 213-229	to explore nurses' experiences of being bullied as well as their beliefs and perceptions of bullying and why it took place	not specified (thematic understanding of the findings)	26 nurses who had experience of WPB (14 held senior clinical positions providing clinical leadership in their specialist field)	in-depth interviews
INT14	M. V. Hutchinson, Margaret H. Wilkes, Lesley Jackson, Debra /Australia	2010	A typology of bullying behaviours: the experiences of Australian nurses	Journal of Clinical Nursing (John Wiley & Sons, Inc.) 19(15-16): 2319-2328	to explore the nature of bullying in the Australian nursing workplace	content analysis	26 nurses who had experienced bullying from two Australian area health services	in-depth, semi-structured interviews
INT15	S. L. Johnson /U.S.A	2019	Workplace bullying, biased behaviors and performance review in the nursing profession: A qualitative study	Journal of Clinical Nursing (John Wiley & Sons, Inc.) 28(9/10): 1528-1537	to explore staff nurses' discourses of WPB and critically examine how these discourses affect their responses to bullying	Foucauldian critical discourse analysis (CDA)	13 staff nurses (12 female, 1 male)	interviews
INT16	Y. M. C. Leong, J. /Singapore	2016	Tough love or bullying? New nurse transitional	Journal of clinical nursing 25(9-10): 1356-	to explore how new nurses experienced role transition in Singapore during their first year	constructivist grounded theory	new nurses with at least 2–3 months of clinical experience (n=26) and	semi-structured interviews and reflective journal entries (new

			experiences	1366	in the clinical setting<br>central research question guiding the study: what factors influence the orientation and transition experience of newly qualified and inexperienced nurses into the professional role?		preceptors (n=5) from 5 different hospitals	nurses only)
INT17	K. M. H. Lux, J. B. Peden, A. R.<br>/U.S.A	2012	Successful management of disruptive behavior: A descriptive study	Issues in Mental Health Nursing 33(4): 236-243	to describe techniques nurses use to successfully manage disruptive behavior (DB) of colleagues	qualitative description, content analysis	9 RNs who had successfully managed DB working at 3 hospitals in OH state (clinical experience 10–44 years, age 32–62)	one-time in-depth interview
INT18	C. I. MacKusick, P. Minick<br>/U.S.A	2010	Why Are Nurses Leaving? Findings From an Initial Qualitative Study on Nursing Attrition	Medsurg Nurs 19(6): 335-340	to understand the factors influencing the decision of RNs to leave clinical nursing	interpretive phenomenological analysis	10 licensed RNs with a minimum of 1 year of clinical practice and no clinical practice in the last 6 months (1–18 years, M=6.5, SD=5.1, female 8, male 2)	semi-structured interview & field notes
INT19	B. H. Mammen, D. J. Lam, L.<br>/Australia	2018	Newly qualified graduate nurses' experiences of workplace incivility in Australian hospital settings	Collegian 25(6): 591-599	to explore NGNs' experiences of workplace incivility while enrolled in graduate nurse programs	descriptive-qualitative method, thematic analysis	8 newly qualified graduate nurses who had experienced workplace incivility during their graduate year (female 6, male 2)	face-to-face interviews
INT20	P. B. McPherson, T.<br>/U.S.A	2019	In their own words: Nurses countering workplace incivility	Nurs Forum 54(3): 455-460	to address the existing gap between identified workplace incivility and the need for active interventions to manage the incivility	phenomenology, content analysis	10 RNs currently or previously working in an acute setting	interviews
INT21	M. P. Roets, M. Myburgh, C.<br>/South Africa	2018	Psychiatric nurses' experience of aggression amongst colleagues	Health SA Gesondheid 23(0), a1086	to explore and describe how psychiatric nurses experienced aggression amongst colleagues in the work environment	thematic coding (Holloway & Wheeler, 2010)	8 psychiatric nurses exposed to aggression by their colleagues in an academic psychiatric hospital	in-depth phenomenological interviews, observation, field notes
INT22	S. R. M. Simons, B.<br>/U.S.A	2010	Bullying in the workplace -- a qualitative study of newly licensed registered nurses	AAOHN Journal 58(7): 305-311	to explore the stories of bullying among nurses based on actual or witnessed experiences	content analysis	184 nurses licensed within 3 years; 139 actually experienced WPB, 14 witnessed WPB; age range 22–61	analyzed written narratives from email surveys
INT23	S. S. Simons, P.<br>/U.S.A	2013	An exploration of the workplace bullying	Journal for nurses in professional	to explore nurses' perceptions of being bullied at work and strategies that nurses use to cope	conventional content analysis	14 women and 4 men (16 White, 2 Black, 12 staff nurses, nurse	individual interviews

			experience: coping strategies used by nurses	development 29(5): 228-232	with bullying behaviors		educators, quality improvement managers, and case managers), age range 23–62 years	
INT24	A. J. J. Skarbek, S. Dawson, C. M. /U.S.A	2015	A Phenomenological Study of Nurse Manager Interventions Related to Workplace Bullying	The Journal of nursing administration 45(10): 492-497	to examine WPB from the perspectives of NMs who manage inpatient units in acute care hospital settings	phenomenological techniques	6 NMs (5 female, 1 male) from hospital settings with at least 1 year of experience in the role	in-depth, semi-structured individual phone interviews
INT25	R. A. T. Taylor, Steven S. /U.S.A	2017	Enactors of horizontal violence: The pathological bully, the self-justified bully and the unprofessional co-worker	Journal of Advanced Nursing 73(12): 3111-3118	to explore individual nurses' perceptions of horizontal violence in the context of their work environment, the meaning they give to the phenomenon and its associated behaviors, as well as the language used to describe the phenomenon	qualitative, descriptive study	observation: 120 employees (80 RNs, 22 patient care assistants, 14 unit secretaries, 2 nurse educators, & 2 NMs)<br>interviews: 31 nurses	observation (370 hours, 2 inpatient units), document review (facility's WPB policy and facility's Code of Ethics and Business Conduct), and semi-structured interviews
INT26	R. K. Tuna, B. /Turkey	2019	Workplace bullying: A qualitative study on experiences of Turkish nurse managers	Journal of Nursing Management 27(6), 1159-1166	to determine whether WPB by subordinates and superiors is present among hospital ward NMs and, if so, to identify its causes and impact	qualitative content analysis	25 NMs who had at least 2 years of experience in a hospital ward and led the inpatient units in Istanbul (all female)	individual, in-depth, semi-structured, face-to-face interviews
INT27	S. A. Vagharseyyedin /Iran	2016	Nurses' perspectives on workplace mistreatment: A qualitative study	Nursing & Health Sciences 18(1): 70-78	to report the perspectives of a sample of Iranian nurses concerning workplace mistreatment	conventional content analysis	15 nurses with at least 2 years of nursing work experience, at least a baccalaureate degree in nursing, experience of workplace mistreatment during the past year, and a willingness to participate in the study (nurses in managerial positions were excluded)	semi-structured interviews
INT28	L. A. P. Wolf, C. Clark, P. R. Moon, M. D. Zavotsky, K. E. /U.S.A	2018	Workplace bullying in emergency nursing: Development of a grounded theory using situational	International emergency nursing 39: 33-39	to explore and develop a substantive theory of WPB as it manifests specifically in the emergency department (ED) setting using a modified grounded theory approach	Clarke's situational analysis model	43 emergency RNs (female 37, male 6) (both staff nurses and those who were in administrative positions)	4 focus group interviews

			analysis					
K1	SY Lee, SY Oh, KM Sung	2013	The Experiences of Turnover Intention in Early Stage Nurses	Journal of East-West Nursing Research, 19(2), 168-176	to explore the turnover intention of novice nurses	content analysis	245 nurses with less than 3 years of experience at 6 general hospitals	individual survey with open-ended questions
K2	JS Kang	2017	A qualitative research on turnover experience among hospital nurses	Asia-pacific Journal of Multimedia Services Convergent with Art, Humanities, and Sociology, 7(4), 751-768	to identify nurses' actual turnover reasons and to understand nurses' turnover experiences	content analysis	8 nurses who had experienced turnover at a hospital at least once	face-to-face in-depth interviews
K3	GL Kim, HJ Lee, YS Cho, MH Kim	2013	The Experience of Turnover Decision Making in New Graduate Nurses	Journal of Qualitative Research, 14(1), 23-33	to describe experience of turnover decision making in NGNs	constant comparative method	15 NGNs who resigned from tertiary hospitals within 1 year after first hospital employment	individual interviews
K4	SA Kim, HW Jeon	2014	Experience of Turnover in New Nurses	Journal of Korean Public Health Nursing, 28(3), 644-657	to understand the experience of early turnover of new nurses	phenomenological method	5 new nurses with less than 1 year of clinical experience in clinical practice	in-depth interviews
K5	YM Kim, SN Kim	2016	The convergence study of Experience of Turnover in new graduate nurses within one year	Journal of the Korea Convergence Society, 7(4), 97-106	To explore the experiences of new nurses who have resigned within one year	Strauss Corbin's grounded theory methodology	5 new nurses who resigned within 1 year	in-depth interviews
K6	JY Bae	2016	Experiences of Clinical Nurses in Overcoming Conflicts With Fellow Nurses	Ph. D. Thesis, Kosin University	to develop a substantive theory of the process by which clinical nurses overcome conflicts with fellow nurses	grounded theory	nurses with more than 1 year of work experience at a general hospital	individual in-depth interviews
K7	YS Lee	2007	The Lived Experience of the Burnout of Nurses Working in Cancer Wards	Ph. D. Thesis, Kosin University	to understand the lived experience of burnout of nurses working in cancer wards	hermeneutic phenomenological human science method	9 nurses who worked at least 1 year in cancer wards	in-depth personal interviews
K8	YO Lee, JY Kang, SY Yun, YH Lee, BJ Kim	2013	A Methodological Triangulation Study on the Experience of Horizontal Violence in	Journal of Korean Critical Care Nursing, 6(2), 37-50	to investigate the experience of horizontal violence in intensive care unit (ICU) nurses	content analysis	134 ICU nurses from 5 hospitals	focus group interviews

			Intensive Care Unit Nurses					
K9	YH Lee	2012	A Study on Phenomenological Research of Change Job Experiences of Nurses in Emergency Room	M. S. Thesis, The Graduate School of Public Administration Kyung Hee University	to research the experiences and substantive meanings of changing jobs for nurses who work in emergency rooms	Colaizzi's method	5 nurses who worked in emergency rooms for more than 1 year and changed jobs or resigned	in-depth interviews
K10	MJ Oh	1997	A Study On The Experiences of Turnover among Hospital Nurses	Journal of Korean Academy of Fundamentals of Nursing, 4(2), 193 - 216	to understand the structure of the experiences of turnover among hospital nurses	Van Kaam's method of phenomenology	16 hospital nurses who experienced turnover one or more times	unstructured interviews
K11	YJ Lee, EJ Lee	2014	Conceptual Development of Workplace Bullying: Focusing on Hospital Nurses	Korean journal of health education and promotion, 31(1):57-70	to build a conceptual framework of bullying in the nursing workplace	Strauss & Corbin's grounded theory methodology	14 nurses who experienced bullying at work	in-depth interviews
K12	SH Choeng, IS Lee	2016	Qualitative Research on Nurses Experiencing Taeoom	Korean Journal of Occupational Health Nursing, 25(3):238-248	to describe the experiences of nurses suffering *Tae-um*	phenomenological method	11 nurses who had experienced *Tae-um*	individual interviews
K13	SH Cheong	2018	A Ground Theory on Occurrence and Persistence of Tae-um among Nurses on the Nursing Unit Environment	Ph. D. Thesis, Seoul National University	to explore and interpret the occurrence and continuation of *Tae-um* in the nursing work environment	grounded theory	20 Korean female nurses	1–2 rounds of qualitative interviews through phone, e-mail, and online communication
K14	SH Lee, SE Chung	2007	The Experience of Verbal Violence between Nurses	Journal of Qualitative Research, 8(1), 79-89	to identify nurses' experiences of verbal violence from other nurses	Colaizzi's phenomenological method	12 nurses currently working at A hospital located in C city	document analysis, observations, and tape-recorded in-depth interviews
K15	SH Moon	2016	The experiences of anesthesia nurse's conflict	M. S. Thesis, Hanyang University	to explore the conflict that clinical anesthesia nurses experience at work	Colaizzi's phenomenological method	9 anesthesia nurses working at tertiary hospitals in Seoul	in-depth interviews
K16	SI Choi	2016	Analysis of Experiences of Generational Issues among Hospital Nurses	Ph. D. Thesis, Seoul National University	to explore the experiences of generational issues among hospital nurses comprehensively	qualitative thematic analysis method	39 staff nurses from various age groups working in 5 acute care hospitals with more than 600 beds (male	focus group interviews

			Application of Focus Group Interviews				nurses, NMs, and new graduates with less than 1 year of experience were excluded)	
K17	SJ Yoon	2015	Experience in establishing the relationship of new nurses and senior nurses	Ph. D. Thesis, Kangwon National University	to understand and describe the relationship experiences of senior and new nurses within the hospital organization	Colaizzi's phenomenological method	14 new nurses with less than 1 year of experience and 16 senior nurses with more than 6 years of experience at the different units of university hospitals with around 500 beds	in-depth interviews
K18	YO Jeoung, SC Park, JK Jin, JY Kim, JU Lee, SY Park, SH Sok	2014	Content Analysis of Communication between Nurses during Preceptorship	Journal of Korean Academy of Psychiatric and Mental Health Nursing, 23(2), 89-92	to explore communication between nurses during preceptorship	qualitative study using content analysis	10 nurses working in urban hospitals	semi-structured interviews
K19	YH Won, JY Kang	2014	Intensive Care Unit Nurse's Communication Experience	Korean Journal of Adult Nursing, 26(3), 352-361	to explore the communication experience of ICU nurses in the workplace	Colaizzi's phenomenological method	15 ICU nurses from two university hospitals	focus group interviews
K20	YO Jeong	2014	Content analysis of communication between nurses - Focused on handoff communication -	Master's thesis, Kyung Hee University	to identify the real state and problems of nurses' handoff communication	qualitative content analysis	10 female nurses aged 27–44 years who worked at general hospitals in Seoul and Gyeonggi-do	in-depth, semi-structured interviews
K21	KO Park, JK Kim	2013	A Study on Experience of Transition from New Clinical Nurse to Competent Step	The Journal of Korean Academic Society of Nursing Education, 19(4), 594-605	to describe nurses' experiences in the transition from NGNs to professional clinical nurses in a hospital	Colaizzi's phenomenological method	10 new clinical nurses working in a hospital	in-depth interviews
K22	JH Kim, JS Lee	2013	The Experience of Clinical Nurses' Interpersonal Conflict	Journal of Qualitative Research, 14(1), 70-80	to determine the meaning and the substance of clinical nurses' interpersonal conflict	Colaizzi's phenomenological method	7 nurses working in general hospitals in Seoul	in-depth interviews
K23	HS Park, KN Kim, EH Kang, JM Lee, SM	2011	Lived Adaptation Experiences of New ICU Nurses	Journal of the Korean Academy of	to determine the meaning of the adaptation experiences of new ICU nurses who were working	Colaizzi's phenomenological method	6 new nurses who had worked for less than 1 year in a newly	in-depth interviews

	Park		Who are Working in a Newly Established University Hospital	Fundamentals of Nursing, 18(2), 226-236	in a newly established university hospital		established ICU of a university hospital located in Y city	
K24	YS Byeon, MY Kim	2009	Interpersonal Conflict Experiences of Nurses	Journal of Qualitative Research, 10(2), 142-151	to understand the difficulties and conflicts that nurses working in clinical institutions experience in interpersonal relations	Colaizzi's phenomenological method (1978)	10 nurses working in tertiary hospitals	unstructured interviews
K25	BM Im, JM Park, MJ Kim, SU Kim, JH Maeng, LL Lee, KA Kang	2015	A Phenomenological Study on the Turnover Experience of Novice Nurses Working in General Hospital	Korean Journal of Occupational Health Nursing, 24(4), 313-322	to identify novice nurses' experience of turnover	Van Kaam's method of phenomenology	12 novice nurses with turnover experiences	in-depth interviews and e-mail communications
K26	YO Suh, KW Lee	2013	Lived Experiences of New Graduate Nurses	Journal of Korean Academy of Nursing Administration, 19(2), 227-238	to explore the lived personal and clinical experiences of NGNs during their first year	Giorgi's phenomenological method	11 NGNs	in-depth interviews and participant observation
K27	JY Kang, SY Yun	2016	A Grounded Theory Approach on Nurses' Experience with Workplace Bullying	J Korean Acad Nurs, 46(2), 226-237	to explore the WPB experiences of Korean nurses	Corbin and Strauss's grounded theory method	20 current or former hospital nurses who had experienced WPB	focus group and individual in-depth interviews
K28	DW Kwon, BS Lee	2018	Experience of the Clinical Nurse 'Taeoom' Recognition of Nurses and Fellow Physicians	The Journal of Humanities and Social science, 9(4), 581-596	to understand, describe, and explain clinical nurses' experiences of *Tae-um* from fellow nurses and physicians	qualitative content analysis method of Graneheim & Lundman (2004)	18 nurses and 5 physicians working at general hospitals in Seoul, Daegu, and Pohang	individual interviews
K29	SY Kim, KO Park, JK kim	2013	Nurses' Experience of Incivility in General Hospitals	Journal of Korean academy of nursing, 43(4), 453-467	to describe nurses' experience of incivility in hospitals and consider their work environment	Colaizzi's phenomenological analysis	7 experienced clinical nurses working on a general ward (2 nurses), ICU (2 nurses), emergency room (2 nurses), or operating room (1 nurse)	in-depth interviews
K30	KH Yi	2017	Construction and Contextualization of Nurses'	Master's thesis, Seoul National University	to explore the constructions and the contextualization of hospital nurses' organizational silence	grounded theory	17 frontline staff nurses	in-depth interviews

			Organizational Silence in Hospitals					
K31	SM Han	2019	The Uncomfortable Experiences in Relationships among Hospital Nurses	Master's thesis, Chuncheon; Hallym University	to understand the discomfort hospital nurses experience in their relationships with other nurses	Colaizzi's phenomenological method	10 nurses who had worked in general hospitals for more than 3 months	in-depth interviews
K32	OY Wi	2019	Lived Experience of Workplace Bullying in New Nurses : A Parse Research Method Study	dissertation, Cheonnam National University	to identify the structure of WPB and suggest an esthetical nursing strategy for "true presence" in new nurses	Parse's research method	10 nurses	in-depth interviews, observations, and poems written by the participants
K33	MH Kim	2019	An Exploration of the Workplace Bullying among Newly Graduated Nurses who Left Nursing	The Graduate School of Converging Clinical and public Health Ewha Womans University	to explore the experience of bullying described by NGNs who left their nursing jobs	content analysis	11 NGNs who left nursing	individual in-depth interviews
K34	JH Ha	2019	A content analysis of 'Taeoom' in the view of an incumbent nurse	The journal of health science, 19, 107-117	to understand and describe the *Tae-um* of nurses	qualitative content analysis	40 currently working RNs (all female)	individual survey with open-ended questions
K35	JS Kim	2019	Concept Analysis of nurses' Tae-Um by Hybrid Model	Master's thesis, Chosun University	to identify and clearly define the attributes of *Tae-um* publicly practiced among nurses	grounded theory suggested by Strauss and Corbin (1991)	15 female nurses who have more than 3 months of experience in general hospitals, consider themselves *Tae-um* victims, and have sufficient explanations for *Tae-um*	in-depth interviews

*RN: registered nurse; NM: nurse manager; RPN: registered practical nurse; NGN: new graduate nurse
