# Supplementary material for: A Qualitative Meta-Synthesis of Studies on Workplace Bullying among Nurses
Source: Int J Environ Res Public Health. 2022 Oct 29;19(21):14120. doi: 10.3390/ijerph192114120 (PMC9659011; doi:10.3390/ijerph192114120)
Supplement: Supplementary file 1 [file ijerph-19-14120-s001.zip › Table S2_FINAL.pdf]

Table S2. Examples of analysis of the qualitative studies on workplace bullying among clinical nurses

ID	Characteristics	Types	Recipients (subjects)	Perpetrators (actors)	Instigating conditions	Response to WPB	Mitigating conditions	Consequences
INT1	▪ Bullying behaviors characterized by personality of WPB perpetrators	▪ Non-verbal tactics as a way of belittling their targets.	▪ Those who were likely to avoid conflict and be unable to voice to protect themselves; considered as reserved and weak<br>▪ Older but less experience colleagues	▪ Those who need to dominate, create conflict in order to exert power over victims, no fear of continuing WPB	▪ Power imbalance<br>▪ Contextual opportunities<br>▪ Changing healthcare work environment<br>▪ Ineffective nurse leadership: indifference to intervene resolving conflict or lack of capacity to deliver effective intervention of WPB<br>▪ Workplace cultures that ignore, condone, or even fosters WPB	▪ Remain silent bystanders and chose not to report WPB that they experienced or witnessed as a coping strategy	(suggestions)<br>▪ Ethical leadership to create a positive, productive work environment<br>▪ Open communication that encourage group discussion without focusing on an individual psychological safety encouraging to speak up<br>▪ Fostering teamwork among nurses	▪ Negative impacts on individual health (emotional health and well-being, sadness, depression, internalization of anger), nursing competence (being denied opportunities to learn and practice new skills), organizational health (absenteeism, turnover), patient care (poor communication between nurse colleagues)
INT3	▪ Inter-professional conflict, nurse-to-nurse conflict on the job<br>▪ Triggered by differences in race, age, work proficiency (i.e., expertise, knowledge, etc)	▪ Socially excluding victims, discounting their knowledge.	▪ Visible minority and younger nurses	▪ White Canadian nurses	▪ Generational differences in work ethics encouraged bullying behaviors (work to live vs. live to work)<br>▪ Closeness and connectedness of nursing teams that easily escalate conflicts	▪ Coping: try to grow a 'thick skin', ignore incivility, requesting transfer, working and self-developing hard not to be ignored, reporting issues to nurse managers<br>▪ Tendency not to invoke rules and regulations to eliminate WPBs unless the conflicts and abuse get serious	▪ Seeking supportive colleagues, standing up for themselves, and demonstrating competence	▪ Decrease in job performance; nursing effectiveness, teamwork considerable stress to aggravate turnover, absenteeism<br>▪ Negative impact on nurses' health and work, the organization, and patients
INT 16	▪ Tough love'--supervisor strategy, a metaphorical euphemism for workplace bullying<br>▪ Used to condition targets into conforming to expected professional or organizational behavior<br>▪ Negative behaviors of senior nurses towards new nurses, that is rationalized as a well-intentioned but nevertheless abusive strategy ; tough love and its attendant behaviors	▪ Pernicious form of supervision; scolding, discouraging questions, providing predominantly negative feedback	▪ New nurses	▪ Senior nurses	▪ Influence of national culture deeply influenced by Confucian ideals, Chinese-related values, and Singaporean culture to expect compliance in hierarchical relationships<br>▪ An endemic, tolerated aspect of the organizational and professional culture, perpetuated across generations	▪ Keeping quiet, do not talk back<br>▪ Making careful decisions about who to confide in and who to trust<br>▪ Reflection, maintaining a positive attitude, disassociation from the immediate emotional effects of scolding and refocusing on higher objectives	▪ Seeking support from family and friends	▪ Great emotional distress to targets, manifesting as feelings of low self-esteem, anxiety, stress, depression and disempowerment, defenselessness.<br>▪ Damages the transitional experience of new nurses; has the potential to influence decisions on whether to remain in a health organization and indeed the profession.

	as a strategy to facilitate effective learning during transition.							
INT 25	▪ Horizontal violence, defined as any hostile, aggressive and harmful behavior by a nurse or a group of nurses towards a co-worker or group of nurses via attitudes, actions, words and/or other behaviors<br>▪ Individual's personality (defects) or situation<br>▪ The majority of the participants attributed it most often to personality and occasionally to the situation	▪ Repeated, aggressive acts involving power and control over a target or a victim	▪ Preceptee, new graduates	▪ Preceptor, manager,<br>▪ Types of bully: pathological bully, the self-justified bully and the unprofessional co-worker<br>▪ Someone who makes you do their work for them	▪ Nurses do not recognize the phenomenon when they witness or experience it, contributing to its perpetuation<br>▪ Nurse participants were not aware of the details of the facility's policies and procedures related to behavioral expectations		▪ Proactive, not retroactive, administrative leadership in addressing bullying and related behaviors system-wide, engaging, and empowering employees to do the same.	
INT 26	▪ Repeated hostile behaviors by a person or group to another person<br>▪ Individual issues such as dissatisfaction, jealousy, prejudice)		▪ Nurse Managers	▪ Staff nurses (subordinates), a particular or group of nurses, superiors	▪ Increased workload /inappropriate staffing<br>▪ (lack of) leaders' commitment to problem-solving	▪ To remain silent: 'silent phenomenon' about bullying so as to avoid conflict<br>▪ Trying to communicate/explain and convince others; change the unit or institution<br>▪ To move to another unit to escape the problem.		▪ Loss of self-confidence, disrupt motivation, burnout, stress, insomnia, intention to leave or deciding to leave, feeling stronger
INT 28		▪ Aggression (verbal assault, false accusation, unjust discipline, heavy assignment, intimidation, derogatory language)<br>▪ Exclusion (withholding information, support, assistance, and guidance/ selective reporting)	▪ Colleague nurses	▪ Colleague nurses, senior nurses, charge nurse, manager, patients, hospital admin/leadership, etc.	▪ Emotional workload, ED conditions (crowding)<br>▪ Workplace culture to tolerate bullying	▪ Three types of subjects' coping: guilty bystander (the most vulnerable), managing the status quo, calling it out (experiencing less WPB)		▪ Form the boundaries of safe patient care provision, creating groups among the ED staff that were either inside or outside of those boundaries<br>▪ Inadequate clinical readiness, errors, delays in care, substandard care<br>▪ Poor retention, unhealthy work environment, emotional exhaustion
K8	▪ Repeated harassment by a specific co-worker for no reason	▪ Verbal violence: scolding in the wrong way	▪ New nurses	▪ Senior nurses	▪ Vertical peer relationships by length of experience<br>▪ Psychological burden that puts pressure on both parties: juniors	▪ Patience considering various circumstances; taking WPB in a positive way	▪ Relying on each other to endure among nurses in similar situations	▪ Psychological withdrawal: initial efforts to accept WPB as a positive process for work adjustment and self-

	▪ Accusation attributed to the same person for any tasks<br>▪ Harassment through intentional repetition of behaviors that the target dislikes	▪ Controlling the ward atmosphere, urge to resign, intentional insult, backstabbing, disruption with work, ignoring, marginalizing, attacking for non-work-related matters, etc.<br>▪ Physical attacks			who feel pressure to learn work quickly from seniors and seniors who have victim mentality that juniors put them in trouble<br>▪ Aggravated work atmosphere that makes other nurses unnecessarily anxious<br>▪ Bullying the victim, making non-victim nurses conform to the bullying to avoid becoming victims themselves	▪ Take WPB less seriously over time<br>▪ Consideration or execution of a job shift when failure to improve or exposure to excessive horizontal violence.		development, gradually leading to identity confusion, low self-confidence, and consciousness of other people's viewpoints on everything<br>▪ Feeling emotionally upset, angry, anxious, or fearful<br>▪ Experiencing physical symptoms: indigestion, headache, sleep disturbances, etc.
K11	▪ Persistent spread of negative energy in the organization	▪ Persistent verbal and non-verbal harassment, work-related harassment and external threats, alienation	▪ New nurses	▪ Senior nurses (unstable ego level)	▪ Negative conformity network created due to the power imbalance between nurses<br>▪ Authoritative and strict hierarchical culture<br>▪ Ineffective organizational culture including unstable organizational systems and fixed mindsets<br>▪ Ambiguity of responsibility<br>▪ Performance-oriented atmosphere<br>▪ Justified harassment through the use of formal and informal forces formed in the legitimate empowerment process within the organization			▪ Physical and psychological withdrawal<br>▪ Decreased work efficiency<br>▪ Increased distrust of managers<br>▪ Increased intent to leave<br>▪ Defensive nursing care; distrust of ' nursing behavior by patients
K27	▪ Intentional, pre-planned disruptive behaviors toward a specific person as a target<br>▪ Promoted or condoned bullying by multiple nurses<br>▪ Continued process until a new target is determined<br>▪ WPB as a mandatory process of adaptation for everyone	▪ Verbal and physical violence, nonverbal harassment<br>▪ Disadvantages at work: unfair division of work, deferred responsibility, nitpicking, disgracing<br>▪ Psychological violence: invasion of privacy, stigmatization as a problematic nurse, ignoring, and creation of atmosphere that urge to resign	▪ those who do not belong to a clique in the ward<br>▪ people with a passive personality and poor relationship formation		▪ Pressure of work<br>▪ Poorly structured education<br>▪ Blurry boundaries of work<br>▪ Harassment reinforced by tense work characteristics and rigid and hierarchical organizational culture of the ward; Facilitation or overlooking of WPB by nurse managers or fellow nurses<br>▪ An atmosphere in which "NO" cannot be said due to the firmly established hierarchical relationship depending on years of experience	▪ Use of a strategy to avoid being hurt by viewing WPB as a stimulant for one's own development<br>▪ Studying in advance and working harder for recognition<br>▪ Use of strategies tailored to the bully<br>▪ Consideration of intent to leave when failure in various attempts or strategies to overcome WPB or actual resignation<br>▪ Relief of tension in work relationships through private meetings or promoting ties leading to a change to two-way communication	▪ Support of victims by a preceptor or nurse manager<br>▪ Seeking empathy and advice from seniors and peers; the more assistance they receive, the more strategies they tend to attempt<br>▪ Mediation by the nurse manager to resolve the conflict; efforts at the nursing department level to improve the culture for colleagueship	▪ Experiencing both physical and psychological symptoms: heart palpitations, insomnia, nightmares, weight loss, depression, low self-esteem, psychological withdrawal that become reasons for resignation in the future<br>▪ Passiveness is likely to be acquired due to WPB

K31	▪ Repetitive, excessive criticism and reprimand or unwarranted criticism<br>▪ Intentional insult<br>▪ Lack of someone to share the hardship with	▪ Extremely aggressive language and behavior; being unable to communicate<br>▪ Physical violence<br>▪ Stigmatizing: making people feel useless, public disgrace, criticism on appearance<br>▪ Making a partiality, alienation<br>▪ Shifts of duties & responsibilities	▪ Peer nurses	▪ Mainly senior nurses	▪ Military-like hierarchies: vertical hierarchical culture of nursing organizations<br>▪ Standards for higher/lower ranks that change at one's own convenience<br>▪ Lack of leadership; managers who condone WPB, discussion regarding WPB with superiors exacerbates the situation<br>▪ Blurred boundaries of work responsibilities	▪ Efforts to meet the needs of seniors	▪	▪ Psychological stress: fear of stigmatization, unbearable situations, withdrawal, desperation, enduring with unfaireness, loss of willingness, feeling of isolation<br>▪ Physical symptoms<br>▪ Keeping distance from seniors in the workplace
K33	▪ Learning by getting scolded	▪ Physical violence: kicking, pinching, hitting, etc.<br>▪ Rebuke and abusive language in public<br>▪ Creation of an overbearing atmosphere through nonverbal expressions<br>▪ Instruction with intense emotions: ignoring, excluding from duties, and not providing feedback or corrections for errors<br>▪ Disadvantages in work: being deprived opportunities to apply for transfer, payment for overtime, shift change request, etc. only	▪ New nurses	▪ Preceptors	▪ Preceptor's work overload<br>▪ Lack of standardized work guidelines<br>▪ Poor working conditions for nurses in hospital settings<br>▪ Rigid, hierarchical work atmosphere: difficult to raise different opinions due to the clear hierarchical relationship by length of experience<br>▪ Inheritance of WPB: victims become perpetrators through accumulated years of work	▪ Enduring until the situation improves, maintaining silence<br>▪ Personal effort to enhance work proficiency<br>▪ Efforts to improve ties with a preceptor	▪ Diverting attention to hobby activities; soothing psychological difficulties by communication with colleagues through personal social network services	▪ Physical and psychological exhaustion<br>▪ Loss of confidence in nursing work and confusion about the identity of the nursing profession<br>▪ Skepticism about future self through witnessing the inheritance of WPB and painful work relationships<br>▪ Resignation if there is no room for improvement

		because of being a new nurse						
