# Supplementary figures and images for: A Qualitative Meta-Synthesis of Studies on Workplace Bullying among Nurses
Source: Int J Environ Res Public Health. 2022 Oct 29;19(21):14120. doi: 10.3390/ijerph192114120 (PMC9659011; doi:10.3390/ijerph192114120)

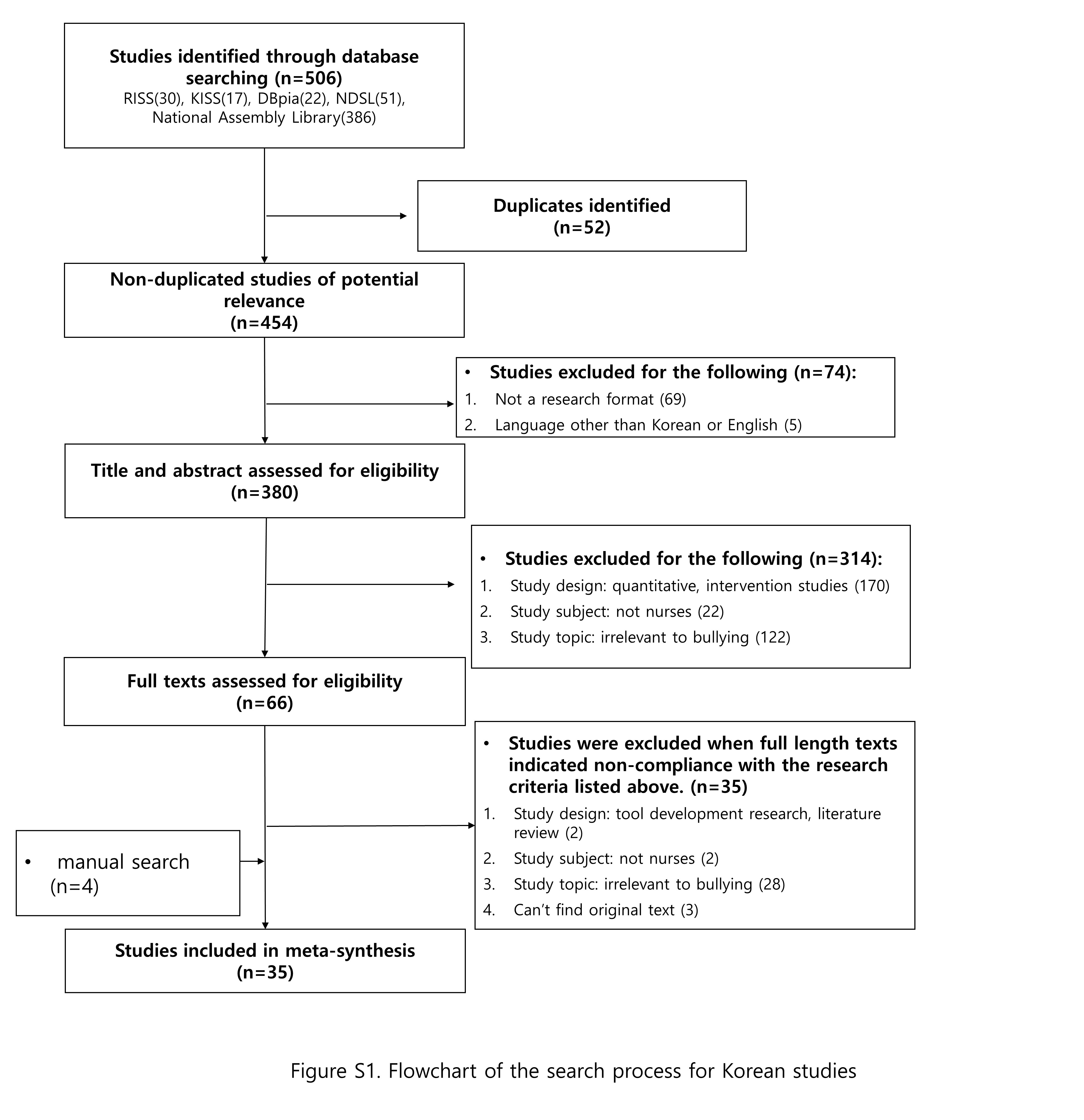

Supplement: Supplementary file 1 [file ijerph-19-14120-s001.zip › FIG S1_R.jpg]

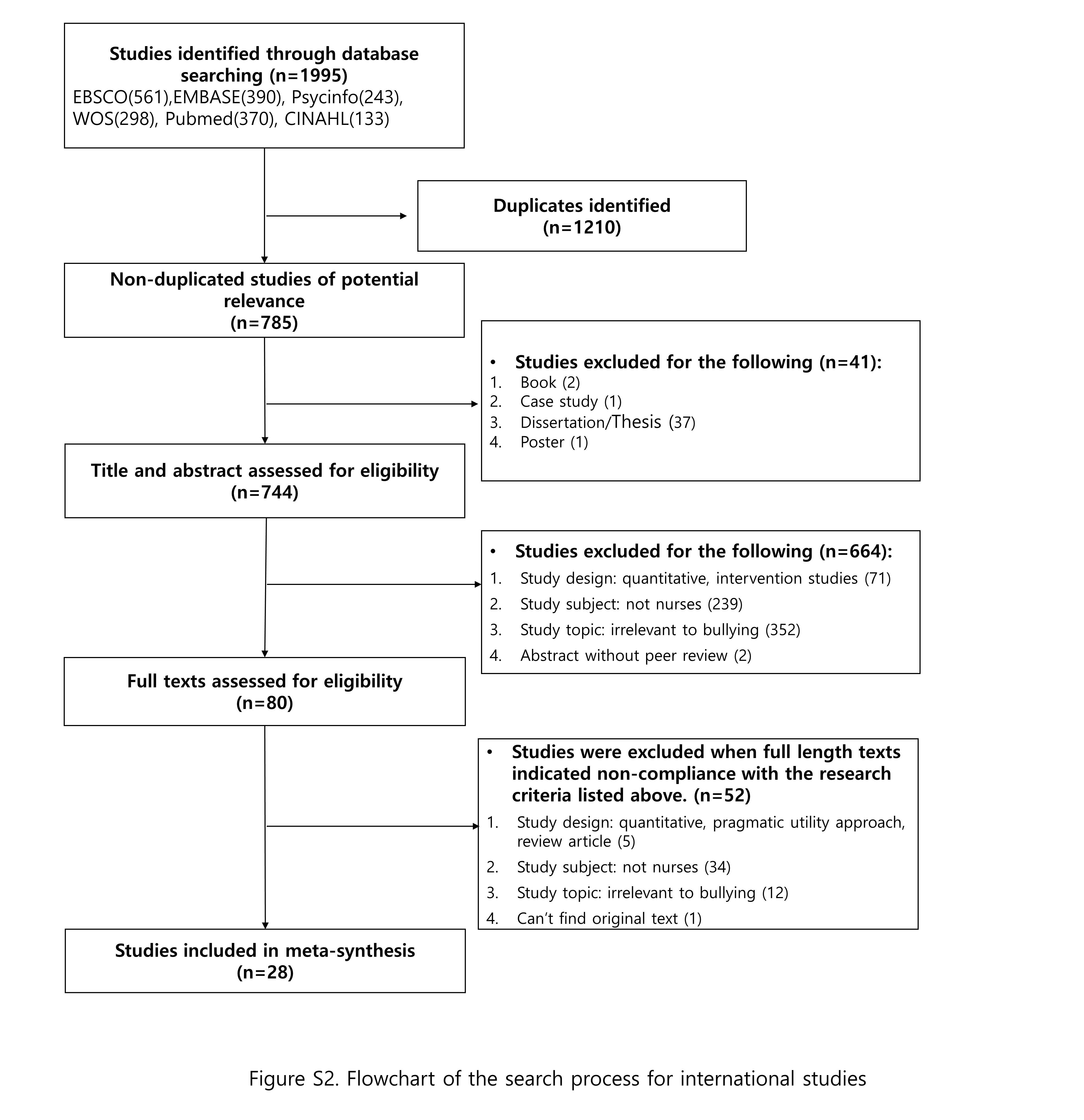

Supplement: Supplementary file 1 [file ijerph-19-14120-s001.zip › FIG S2_R.jpg]
